# Bayesian analysis of home advantage in North American professional sports before and during COVID-19

**DOI:** 10.1038/s41598-021-93533-w

**Published:** 2021-07-15

**Authors:** Nico Higgs, Ian Stavness

**Affiliations:** Computer Science, University of Saskatchewan, Saskatoon, S7N 5A2 Canada

**Keywords:** Mathematics and computing, Computational science, Computer science, Scientific data, Statistics

## Abstract

Home advantage in professional sports is a widely accepted phenomenon despite the lack of any controlled experiments at the professional level. The return to play of professional sports during the COVID-19 pandemic presents a unique opportunity to analyze the hypothesized effect of home advantage in neutral settings. While recent work has examined the effect of COVID-19 restrictions on home advantage in European football, comparatively few studies have examined the effect of restrictions in the North American professional sports leagues. In this work, we infer the effect of and changes in home advantage prior to and during COVID-19 in the professional North American leagues for hockey, basketball, baseball, and American football. We propose a Bayesian multi-level regression model that infers the effect of home advantage while accounting for relative team strengths. We also demonstrate that the Negative Binomial distribution is the most appropriate likelihood to use in modelling North American sports leagues as they are prone to overdispersion in their points scored. Our model gives strong evidence that home advantage was negatively impacted in the NHL and NBA during their strongly restricted COVID-19 playoffs, while the MLB and NFL showed little to no change during their weakly restricted COVID-19 seasons.

## Introduction

In professional sports, home teams tend to win more on average than visiting teams^1–3^. This phenomenon has been widely studied across several fields including psychology^4,5^, economics^6,7^, and statistics^8,9^ among others^10^. While home advantage is now a widely accepted phenomenon, the magnitude of the advantage and its cause are not as clearly understood or widely accepted as its existence. Part of the difficulty in analyzing the specifics of home advantage is due to the lack of controlled experiments, because nearly every professional game is played in one of the team’s home stadium in their home city. While there have existed some show matches at neutral sites, their relative sample sizes are too small from which to draw any reasonable conclusions. For example, the National Football League only plays about 4–5 neutral site games out of a total 256 games each regular season.

The return to play of professional sports during the COVID-19 pandemic presents a unique opportunity to analyze teams playing in situations where home advantage may genuinely no longer apply. The leagues have restricted travel and fan attendance or even created a bubble where only one or two stadiums are used and only the players and necessary staff are present for the games. We consider this restricted return to play as a control group where travel, home stadium familiarity, and home crowd have been controlled (i.e. removed) for enough games to provide a reasonable sample to analyze. There has been considerable academic work analyzing the effect of COVID-19 restrictions on home advantage in European football^10^. However, comparatively there has been a lack of work analyzing the effect in the North American professional sports leagues. In fact, to the authors knowledge there has only been one work focused on home advantage during COVID-19 across the big four North American professional leagues; and it only investigated the NBA^11^. In this work, we aim to fill this gap by inferring the effect of and changes in home advantage prior to and during COVID-19 in the big four North American leagues: the National Hockey League (NHL), the National Basketball Association (NBA), Major League Baseball (MLB), and the National Football League (NFL).

Professional sports leagues adopted different restrictions in response to the COVID-19 pandemic. The NHL and NBA had the strongest restrictions where they both created a COVID-19 bubble where all games were played at the same consistent location with players quarantined together separate from their families and the outside world. While this proved to be extremely effective in terms of player safety^12,13^ it seems likely that it was the most extreme in terms of its effect on players performance and psychology. In contrast, teams in the MLB and NFL still traveled to their opponents home stadiums. These leagues restricted fan attendance and media access, with some NFL stadiums allowing small amounts of fans to attend. Thus, all leagues lacked a potential home crowd effect, but only the NHL and NBA restrictions removed the additional factors of travel and home city familiarity. This is noteworthy because of the implications in relation to previous work investigating the causes of home advantage^2,5,8,11,14–16^. In McHill and Chinoy^11^, the authors argue that home advantage in the NBA’s COVID-19 bubble arose from either circadian disruption or the general effect of travel. Our work builds upon such previous works by considering the NBA’s COVID-19 bubble and its effects on home advantage while also comparing and contrasting to other similar COVID-19 bubbles in the NHL and different COVID-19 restrictions seen in the MLB and NFL.

We adopt a Bayesian framework to develop a Negative Binomial regression model that adjusts for relative team strengths while inferring home advantage. We choose this approach for two main reasons. First, alternative methods that rely on correlations among raw statistics, such as home win percentage, fail to account for other factors such as relative team strengths. Our regression approach can infer changes in team performance while adjusting for quality of opponents. Second, the Bayesian framework gives more interpretable results and more flexibility in model building than classical regression methods. The Bayesian framework results in distributions for the estimates of each parameter in our model. This allows us to analyze these distributions directly to determine the probability a parameter is greater (less) than a certain value or that it exists in a specific interval, avoiding the confusion that often arises interpreting p values and confidence intervals.

By examining the resulting home advantage parameter estimates of our model from before and during the COVID-19 pandemic, we can draw conclusions about the existence of the home advantage phenomenon and provide new evidence for its potential causes. We hypothesize that home advantage is a real phenomenon, thus we expect its parameter estimate to drop during the COVID-19 seasons relative to before the COVID-19 seasons. We are also interested in examining if any differences in relative changes in home advantage exist across the leagues as some leagues had different COVID-19 restrictions which could affect home advantage differently. We also show that point totals in North American professional sports are prone to overdispersion, thus, the Negative Binomial distribution allows for better model fit than the more common Poisson and Normal distributions used in regression analyses.

## Results

**Figure 1:**
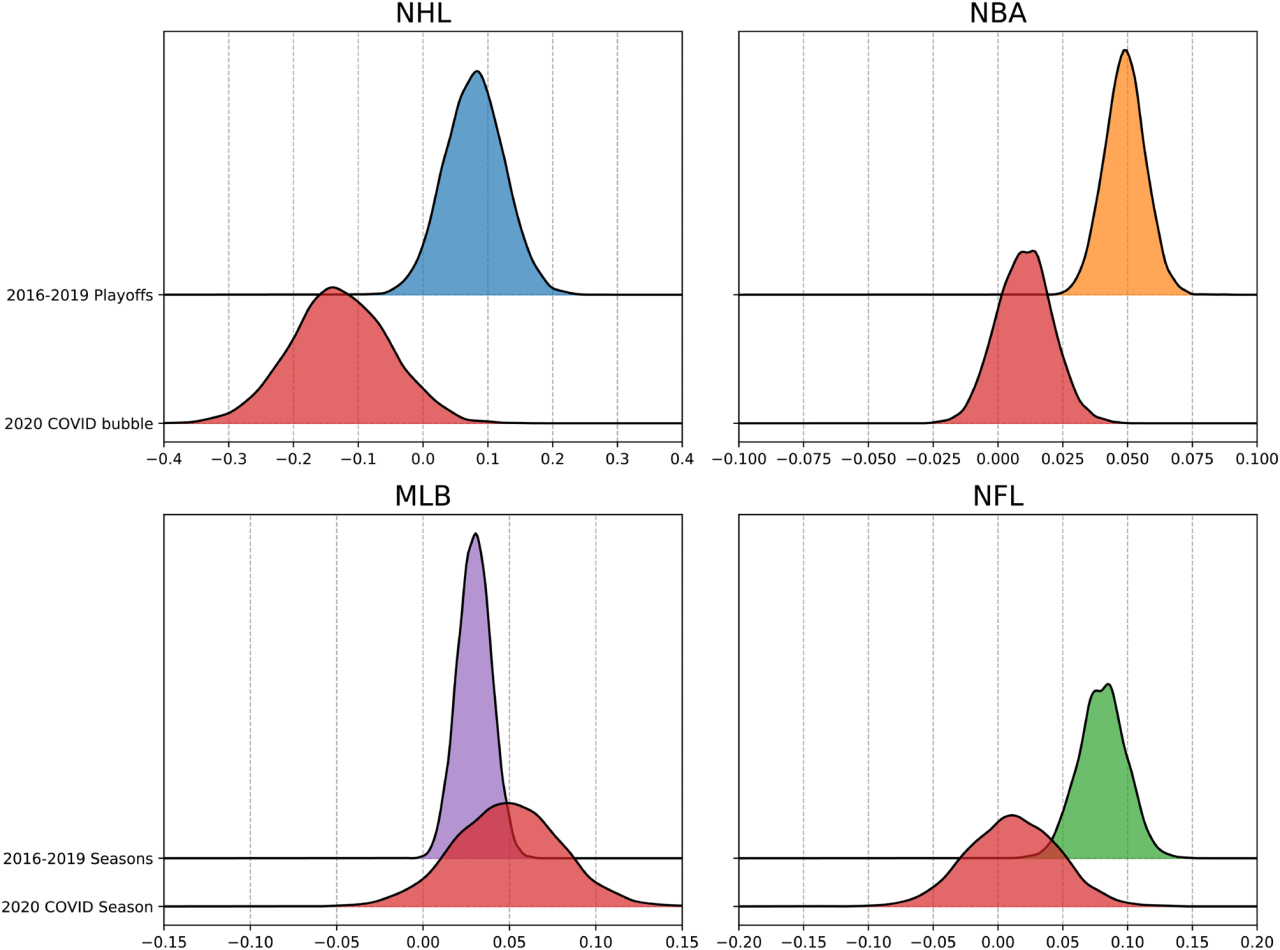
Distributions of the estimated home advantage for the NHL, NBA, MLB, and NFL for pre and post COVID adjusted seasons. Home advantage for playoffs are reported for NHL and NBA because that is when their COVID restricted games took place. Home advantage for regular season is reported for MLB and NFL as their respective playoff seasons are too small for stable results. Red distributions represent COVID-19 bubble adjusted seasons.

The distributions for the estimates of the home advantage parameters from pooling the previous four pre-COVID-19 seasons/playoffs together can be seen in Fig. 1 with the COVID-19 restricted season/playoffs coloured red. The peaks of these distributions represent the most likely values for the home advantage parameter and their width represents the uncertainty in these estimates. We can use these distributions to directly measure the probability the home advantage parameter is less than the previous seasons. The leftward shift of the distribution for the COVID-19 restricted season/playoffs suggests that home advantage decreased in the NHL, NBA, and NFL while not changing for the MLB.

**Figure 2:**
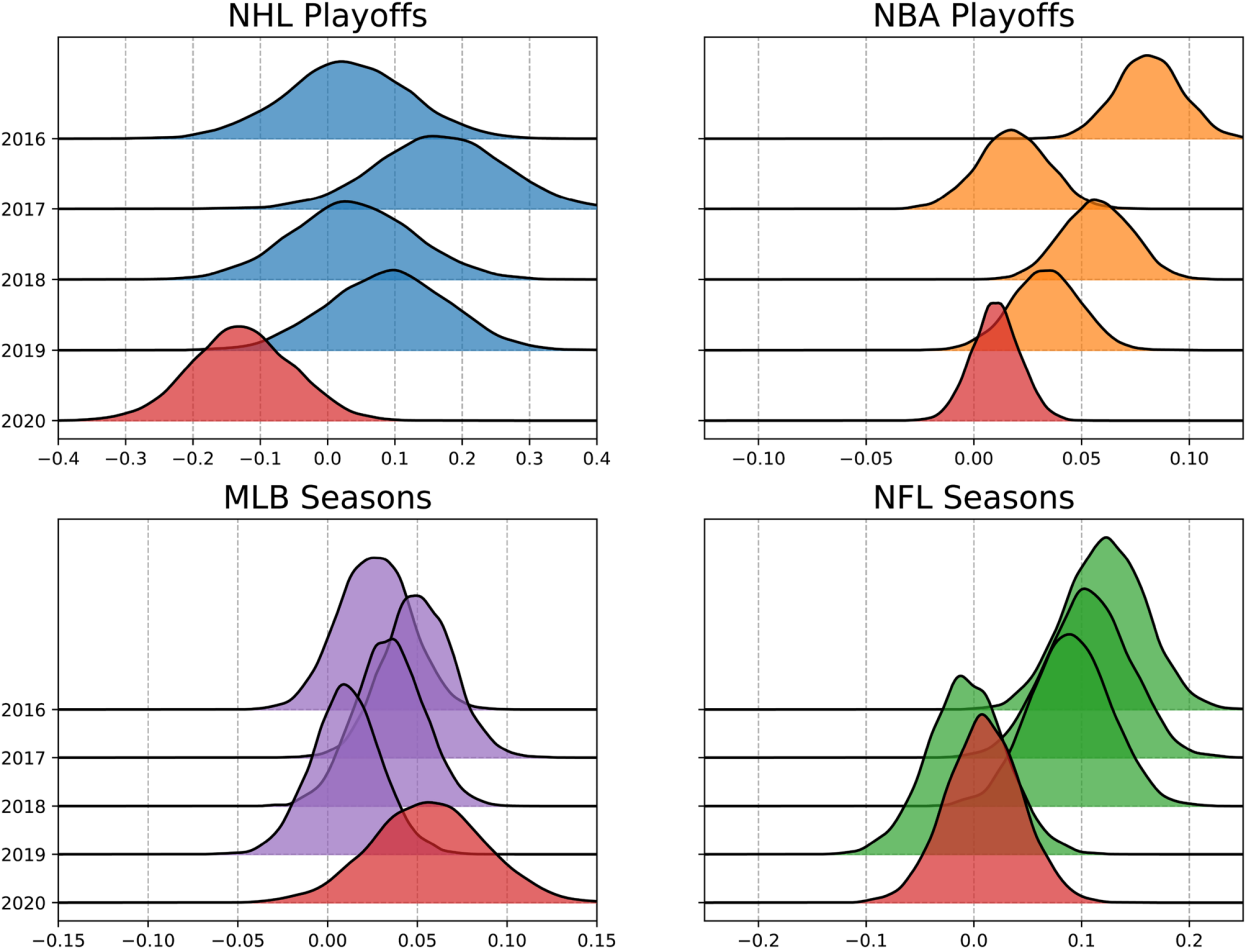
Distributions of the estimated home advantage for the NHL, NBA, MLB, and NFL over the past 5 seasons from 2016 to 2020. Home advantage for playoffs are reported for NHL and NBA because that is when their COVID restricted games took place. Home advantage for regular season is reported for MLB and NFL as their respective playoff seasons are too small for stable results. Red distributions represent COVID-19 bubble adjusted seasons.

Figure 2 shows results from estimating home advantage individually for each prior season. This more granular view of pre-COVID-19 home advantage reveals greater season-to-season variation in home advantage that is missing in Fig. 1. Nevertheless, the year-over-year estimates in Fig. 2 show the results of reduced home advantage in COVID-19 restricted season/playoffs holding for the NHL, NFL, and NBA, albeit with a single past season with lower home advantage in both the NFL and NBA. The remainder of this section examines these estimated distributions and their implications.

For the NHL and NBA data, Figs. 1 and 2 and our analysis focus on their playoff seasons because the NHL and NBA COVID-19 seasons only took place during their playoff seasons. In contrast, the MLB and NFL had COVID-19 restrictions for their entire seasons, therefore, Figs. 1 and 2, and our analysis for those leagues are focused on their regular season games. Focusing on the MLB and NFL regular seasons is not only convenient but arguably necessary as their playoff seasons consist of much fewer games than the NHL and NBA playoff seasons, resulting in high uncertainty of parameter estimates. The NHL and NBA regular season results as well as the MLB and NFL playoff results are provided in the supplementary materials 1.

The home advantage parameter, $\beta$, represents a multiplier of $\text {exp}(\beta )$ applied to expected points. For example, an estimated home advantage parameter for the NBA of 0.05 represents a $\text {exp}(0.05) \approx 1.0513$ multiplier on expected points or an increase in expected points of 5%. With average points scored in the NBA being around 107 this would translate to approximately a 5-point home advantage on average in the NBA playoffs. We provide a full description and interpretation of the model in “Methods” section.

For the NHL data, the results in both Figs. 1 and 2 show the home advantage parameter confidently above 0 for pre-COVID-19 seasons and confidently below 0 for the COVID-19 bubble. The probability the home advantage parameter $(\beta )$ is less than 0 for the COVID-19 bubble is $\Pr (\beta < 0) = 0.95$. The probability the home advantage parameter is less than the previous playoff seasons mean of 0.081 is 0.998. These results give strong evidence that home advantage in the NHL was negatively impacted by the COVID-19 bubble.

For the NBA data, the pooled home advantage parameter estimate in Fig. 1 is confidently above 0 and tightly around 0.05. For the COVID-19 affected playoffs, the probability the home advantage is less than 0 is only 0.17, but the probability that it is less than the pre-COVID-19 mean of 0.05 is 0.999, suggesting that home advantage in the NBA was negatively impacted by the COVID-19 bubble. However, when examining the year-to-year estimates of prior seasons in Fig. 2 we see a decreasing trend in home advantage in the NBA playoffs with the estimate for the NBA playoffs in 2017 appearing as almost as much of an outlier as the COVID-19 estimate. This suggests the decreased home advantage in the COVID-19 could potentially be a random outlier. The uncertainty in these estimates means we can not make definitive conclusions in the absence of more data. We conclude that it is probable that home advantage in the NBA decreased in the COVID-19 bubble but not as definitively as the NHL results.

For the MLB data, the home advantage parameter is surprisingly likely to be slightly greater than it had been in previous seasons. The probability the home advantage parameter is less than the mean of the previous seasons is $\Pr (\beta < 0.036) = 0.26$. When comparing the COVID-19 estimate to the previous seasons in Fig. 2 there appears to be no noteworthy difference. This gives evidence that home advantage in the MLB was unlikely to be negatively impacted by the COVID-19 restrictions and was likely unaffected by the restrictions.

For the NFL data, the pooled home advantage parameter estimate in Fig. 1 is confidently above 0 with a mean of 0.078. For the COVID-19 affected season, the probability the home advantage is less than 0 is 0.388, but the probability that it is less than the pre-COVID-19 mean of 0.078 is 0.976, suggesting that home advantage in the NFL was negatively impacted by the COVID-19 restrictions. However, when examining the year-to-year estimates of prior seasons there is a clear pattern of home advantage decreasing in the NFL and even being lower in 2019 than it was in the 2020 COVID-19 adjusted season. We argue the results in Fig. 2 are enough to overturn the results in Fig. 1 and conclude that home advantage in the NFL was not impacted from its previous trend by the COVID-19 restrictions.

In summary, results for pooled (Fig.  1) and individual (Fig.  2) past seasons give strong evidence that home advantage in the NHL was negatively impacted during the COVID-19 restricted playoff season and that home advantage in the MLB was unaffected by the restrictions. Pooled past season results also suggest home advantage was negatively impacted by the COVID-19 restricted seasons for the NBA and NFL, however a closer examination of the individual past season results reveals a trend of decreasing home advantage over the past few seasons, which may partly account for the lower home advantage found during NBA and NFL COVID-19 restrictions.

### Model fit comparisons

In this section, we present the results of fitting our model with the Negative Binomial distribution as the likelihood for point totals, as compared to the more commonly used Normal and Poisson distributions.

Since point totals in sports are positive integers, the Poisson distribution is a natural choice for modelling their outcomes. The effectiveness of the Poisson distribution for modelling point totals has been shown in several works analyzing European football data^10,17,18^. One shortcoming of the Poisson distribution is that it only has one parameter and this leads to the strong assumption that the mean is equal to the variance. For low scoring sports like European football and hockey, this is usually a fine assumption. However, this is an invalid assumption for several of the sports we analyze in this paper. Table 1 reports the dispersion statistic $\sigma _p$. The dispersion statistic represents how much greater the variance is than the mean while adjusting for sample size and model complexity and is computed as $\chi ^2/(n-p)$ for each league, where $\chi ^2$ is the Pearson chi-squared statistic of the point totals data, and $n-p$ are the degrees of freedom with $n$ representing the sample size of the point totals data and $p$ representing the number of predictors in our model. The commonly suggested threshold, $\sigma _p > T$, for determining when a Poisson model is no longer appropriate is around $1.2< T < 2$^19,20^. Table 1 shows the NBA, MLB, and NFL having potential overdispersion in their point totals and thus, the Poisson distribution is likely inappropriate and less effective. We instead opt for using the Negative Binomial distribution because it has an extra parameter $\alpha$ that gives greater flexibility and better model fit to data that is overdispersed while still adequately fitting models without overdispersion.

To establish the efficacy of the Negative Binomial distribution in our model, we fit and compare models using the Poisson and Normal distributions across each league. We fit Poisson and Normal regression models by changing the likelihood of the model in (1) to $y_{ij} | \mu _{ij} \sim \text {Pois}(\mu _{ij})$ for the Poisson regression (and subsequently drop $\alpha$ from the rest of the model as it is not needed), and $y_{ij} | \mu _{ij}, \sigma ^2 \sim \mathcal {N}(\mu _{ij}, \sigma ^2)$ for the Normal regression (and use a weakly informative prior $\sigma ^2 \sim \text {HalfNormal}(50)$). Otherwise the models are identical and their interpretation remains the same as is discussed in “Methods” section.

We evaluate the models across each league by estimating the out-of-sample predictive fit via leave-one-out cross-validation (LOO). Following the work of Vehtari^21^ we approximate LOO using Pareto-smoothed importance sampling (PSIS) and report the results in Table 1. We note here that we also used the widely-applicable information criterion (WAIC)^22^ but found the results to be nearly identical and the conclusions the same. Examining Table 1 we see that for the NHL and NBA, where there is little to no overdispersion, the Poisson and Negative Binomial models fit almost identically with the Negative Binomial model starting to show small improvement for the slightly overdispersed NBA data. As overdispersion increases for the MLB and NFL data we see the fit of the Negative Binomial model become noticeably better. The Negative Binomial model also outperforms the Normal model across each league except for the NFL where we see it fit only slightly worse while both models greatly outperform the Poisson model.

**Figure 3:**
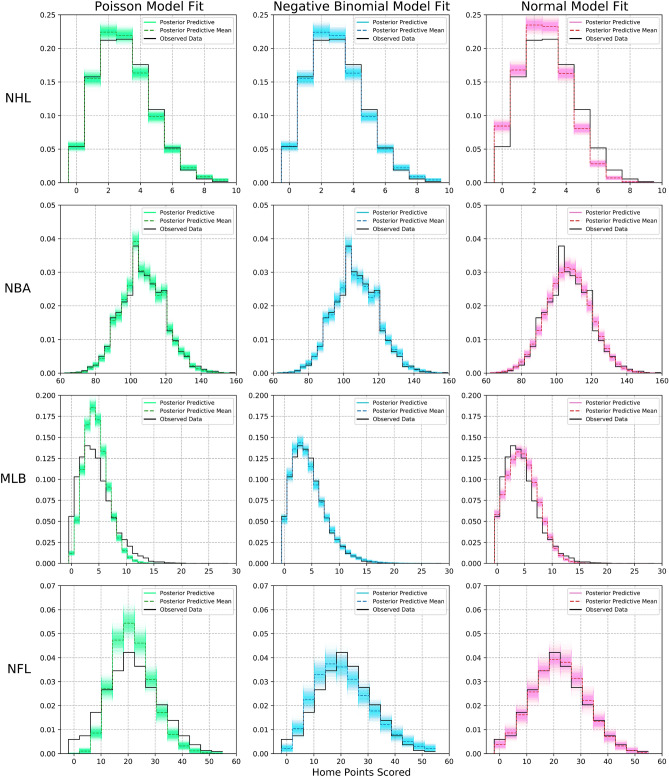
Comparison of distribution of home points in the models and the observed data for each league. The Negative Binomial model noticeably provides a better overall fit across each league.

These differences in fit can be seen visually in Fig. 3 where we plot the distribution of observed home point totals in black along with 2000 sampled model fits in green for Poisson, blue for Negative Binomial, and red for Normal; with the respective mean model fits as dashed lines. The differences between the Poisson and Negative Binomial models becomes increasingly apparent for the leagues with greater overdispersion, while the Normal model comparatively struggles for each league except the NFL where both the Normal and Negative Binomial greatly outperform the Poisson model. Because the point totals of the sports we are considering are positive integers prone to overdispersion and based on the results in Table 1 and Fig. 3, we conclude that the Negative Binomial distribution is the most appropriate for regression modelling professional hockey, basketball, baseball, and American football.

**Table 1:** Comparison of estimated negative log-likelihood of leave-one-out cross-validation (LOO) for each model across each league.

	$\pmb {\sigma _p}$	Model	LOO	dLOO	dSE
NHL	0.99	Poisson	**− 24761.3**	–	–
		NB	− 24761.5	0.2	0.2
		Normal	− 25140.9	379.5	23.4
NBA	1.50	Poisson	− 49018.3	53.5	11.0
		**NB**	**− 48964.8**	–	–
		Normal	− 48981.9	16.6	7.5
MLB	2.27	Poisson	− 57458.7	4115.8	120.9
		**NB**	**− 53342.9**	–	–
		Normal	− 55696.8	2353.17	65.1
NFL	4.56	Poisson	− 11751.2	2042.5	119.0
		NB	− 9841.7	133.0	22.1
		**Normal**	**− 9708.7**	–	–

The bold signifies which model is most likely to have the best predictive performance on unseen data.

The differences between the Poisson, Negative Binomial (NB), and Normal models are reported relative to the best fitting model (dLOO) for each league; along with the standard error of the estimated differences (dSE). The dispersion statistic, $\sigma _p$, indicates how much greater the variance is than the mean for point totals in each league and signals overdispersion when $\sigma _p > 2$. The NB model noticeably outperforms the Poisson model for leagues with greater overdispersion (MLB and NFL) while being nearly identical for leagues with little to no overdispersion (NHL and NBA). The NB model also outperforms the Normal model in each league except the NFL where they are close to one another while both vastly outperforming the Poisson model.

## Discussion

The results of our model when pooling the previous seasons prior to COVID-19 (Fig. 1) show a noticeable decrease in home advantage for the NHL, NBA, and NFL with no noticeable change in home advantage for the MLB. However, while the year-over-year estimates (Fig. 1) corroborate these findings to be significant for the NHL and MLB, they show the results are potentially weaker for the NBA and NFL. We argue that the results in Fig. 2 reveal that home advantage in the NFL was already decreasing leading up to the 2020 season and that the 2020 COVID-19 restricted season had no significant impact on home advantage in the NFL. We further argue that the NBA COVID-19 restricted season may potentially be an outlier similar to the 2017 playoffs. This means we can not be as confident in our conclusions about home advantage decreasing in the NBA as we are with the NHL. We argue the results give evidence that it is likely home advantage decreased in the NBA but we can not be certain with the limited sample we have.

If we contrast the COVID-19 restrictions in the NHL and NBA to the MLB and NFL, there are two notable differences. First, the NHL and NBA had much stricter COVID-19 bubbles where teams did not travel to each others stadiums, whereas the MLB and NFL did travel to the various stadiums and only restricted fans attending. This suggests that the lack of travel and home city familiarity contributes to home advantage more than a home crowd effect, and therefore results in a greater drop in home advantage in the leagues that had a strict bubble compared to the leagues that allowed travel and play at home stadiums. This agrees with McHill and Chinoy^11^ and gives further evidence to the cause of home advantage being more attributable to the general effect of travel. The second difference is the relatively small to no home advantage that the model infers for the MLB and NFL relative to the strongly positive home advantage in recent years found in the NHL and NBA. While we can not fully tease out which of these two differences is stronger, this opens up potential for future work as these leagues continue to play through the COVID-19 pandemic. It will be interesting to see if home advantage returns in the NHL and NBA as they shift toward fewer restrictions similar to the MLB and NFL.

The strongest result for a decrease in home advantage due to COVID-19 restrictions was seen in the NHL. We note that this is particularly interesting because the NHL is somewhat unique to the other leagues, because the home team has an extra difference; they get the last change during stoppages of play, meaning they get to decide player matchups. An analysis of this effect has been carried out by Meghan Hall^23^ who concluded that home teams benefit when they get to control matchups and argued that this benefit should not be discounted during the 2020 COVID-19 bubble season. The results of our model, however, seem to indicate that no home advantage existed during the NHL’s COVID-19 bubble and suggests the effect of last change in the NHL is potentially not as impactful as previously thought.

We have also shown how using the Negative Binomial distribution as the likelihood function for our regression model outperforms the Poisson distribution for sports with overdispersion in their point totals such as the MLB and NFL, while still performing just as well as the Poisson distribution when there is little to no overdispersion such as in the NHL and NBA. We showed the Negative Binomial distribution also outperforms the Normal distribution across all leagues except for the NFL where both models vastly outperformed the Poisson distribution. We argue this is because the Negative Binomial distribution effectively represents positive integers like the Poisson distribution while having an extra parameter, like the Normal distribution, to account for overdispersion which represents a greater spread in the data due to greater variance.

Our Bayesian regression model has three key advantages over traditional methods for inferring home advantage. First, methods that rely on correlations among raw statistics fail to account for factors such as relative team strengths. For example, a weaker team may have a poor home win percentage because they have a poor overall win percentage. That same team; however, may perform better at home than they do at other stadiums whilst still losing to stronger opponents and vice versa. This discrepancy can be further impacted by imbalanced schedules where teams do not face the same opponents as each other in a perfectly balanced manner. While some studies recognize this discrepancy, they often claim that it is a small effect that can be ignored^24^ without showing evidence. We argue that while these claims may hold up for analyses spanning decades they are not appropriate for the short COVID-19 restricted seasons we are considering. Furthermore, these issues and any debate over how much of an effect they have is most reliably mitigated by adjusting for varying team strengths when trying to infer home advantage. Regression analysis methods are primarily used for their ability to account for multiple factors when performing inference, and as such they are most appropriate for our focus of analyzing home advantage. Second, the Bayesian framework gives more interpretable results and more flexibility in model building than classical regression methods. This can be seen in the results of the Bayesian framework being distributions for the estimates of each parameter in our model. In this way, the implied probability and corresponding uncertainty of parameter estimates are still rigorously defined while being directly measurable and more intuitive to understand than traditional Frequentist methods of confidence intervals and p-values. Third, with advancements in computational Bayesian statistics, such as Probabilistic Programming languages^25^ and Hamiltonian Monte Carlo (HMC)^26^, we are able to easily define and compute flexible and complex models using various likelihood functions with ease instead of being limited to traditional methods like Normal and Poisson regressions more traditionally used in sports modelling^9,10,17,18,27^.

While our model has produced some interesting results, it is worth discussing some of its limitations and areas for future work and improvement. The most notable limitation is that the COVID-19 lockout and restricted seasons are unprecedented and come with additional caveats such as protocols for testing, impact of positive tests, reduced practices, and players being away from their families, that extrapolating all results to home advantage or fan impact alone does not address all the possible factors influencing player and team performance. The model also does not account for travel or rest before games as a potential confounding factor for home advantage. This was ignored primarily due to it being irrelevant for the NHL and NBA COVID-19 bubbles, but for the less restrictive MLB and NFL seasons as well as future COVID-19 restricted seasons this could be a potential factor worth exploring. The model could benefit by including group level factors when estimating the offensive and defensive strengths of teams. The multilevel structure of the Bayesian framework we have adopted naturally allows for such inclusions^28–30^. For example, we hypothesize that advanced analytics metrics such as expected goals (xG) and corsi in hockey, regularized adjust plus-minus (RAPM) in basketball, hitter splits and park factors in baseball, yards gained/allowed above/below expected in football, could all be leveraged to improve team strength estimates. This could also include personnel differences such as the effect of star players being injured, back-up goalies starting, or starting pitchers being included in the estimates of a teams relative strength for a given game. These inclusions are beyond the scope of this work as these analytics and personnel changes and their effect differ greatly across different sports. In future work, we hope to focus on an individual sport and include such factors, using the current model as a baseline to compare against. Our model is also limited by focusing on only point totals to infer home advantage, while some previous works also analyze differences in penalties to assess a home advantage in the officiating of games^5,7,8,10^. This was excluded from this work because of how much penalties and their effect differ across the various sports we considered, but is something we hope to explore in the future when analyzing a single sport in more depth.

## Methods

We infer home advantage by fitting a regression model to predict the points scored in each game while adjusting for relative team strengths and home advantage. We adjust for relative team strengths by modelling both an offensive rating and a defensive rating for each team. We argue this better represents real differences between teams and allows the model to better infer if a team performs better or worse when playing at home by measuring its performance relative to its average offensive performance versus its opponents average defensive performance. This section describes in detail the parameters of the model, their interpretation, and how we fit the model.

We aimed to build a parsimonious model to infer home advantage for each league while adjusting for relative team strengths and accounting for uncertainty in the data and parameter estimates. We needed a method that was robust to smaller sample sizes because we only had one COVID-19 adjusted season for each league to compare to and because this sample becomes smaller as you include more parameters which splits the data into smaller groups. We also wanted to be able to quantify the uncertainty in our parameter estimates. To address these concerns we adopt a Bayesian multi-level regression model framework building upon previous work^9,10,18,27^ that allows for pooling results across all teams to infer home advantage. The partial-pooling of multi-level regression modelling allows us to separate the effects of individual teams offensive and defensive strengths from their group level means and helps prevent overfitting by adjusting parameter estimates through a process commonly referred to as “shrinkage to the mean”^28–30^. We argue the pooling of data across each teams results to better handle smaller sample sizes while preventing overfitting, and the ability to quantify the uncertainty in parameter estimates makes Bayesian multi-level regression an ideal choice for this task.

We model the response variable of the number of points scored by each team in each game as Negative Binomial:

1 $$\begin{aligned} y_{ij} | \mu _{ij}, \alpha _{ij} \sim \text {NegativeBinomial}(\mu _{ij}, \alpha _{ij}) \end{aligned}$$

where $y_{ij} = [y_{i1}, y_{i0}]$ is the vector of observed points scored in game $i$ by the home ($j=1$) and away ($j=0$) teams and $\mu _{ij} = [\mu _{i1}, \mu _{i0}]$ are the goal expectations of the home and away teams in game $i$. The $\alpha$ parameter allows for the flexibility of fitting to overdispersed data where the variance is much greater than the mean. In our experiments we have found that defining $\alpha$ as a fraction of $\mu _{ij}$ led to better sampling and model fit. Thus, we define $\alpha _{ij} = \mu _{ij} \times \lambda$ and then sample $\lambda$ when fitting the model. We model the logarithm of goal expectation as a linear combination of explanatory variables:

2 $$\begin{aligned} \begin{aligned} \text {log}(\mu _{i1})&= \gamma _{sp} + \beta _{sp} + \omega _{sh[i]} + \delta _{sa[i]} \\ \text {log}(\mu _{i0})&= \gamma _{sp} + \omega _{sa[i]} + \delta _{sh[i]} \end{aligned} \end{aligned}$$

where $\gamma _{sp}$ is the intercept term for expected log points in season, with $s = [0, 1, 2, 3, 4]$ corresponding to the 2016, 2017, 2018, 2019, and 2020 seasons respectively. The subscript $p$ indicates regular season ($p=0$) or playoffs ($p=1$). For the results in Fig. 1, all previous seasons are combined ($s=0$) and compared to the COVID-19 adjusted season ($s=1$). Home advantage is represented by $\beta _{sp}$ with $s$ and $p$ the same as the intercept. The offensive and defensive strength of the two teams are represented by $\omega$ and $\delta$. The nested indexes $h[i]$ and $a[i]$ identify the teams playing at home and away respectively and we use this nested notation to emphasize the multi-level nature of these parameters as they are modelled as exchangeable from a common distribution^28–30^. This enables pooling of information across games played by all teams in a league and results in mixing of the observable variables $(y_{ij})$ at this higher level which accounts for correlation in home and away points scored in each game^18^.

In this model formulation we are estimating different home advantage parameters for the regular season and playoffs as well as for each individual season. The primary motivation for this is because the NHL and NBA COVID-19 bubbles essentially only occurred during their playoffs and we therefore want to separate home advantage during the playoffs for a more direct comparison. Modelling in this way also addresses potential questions of whether home advantage changes each year or remains constant. Our results in Fig. 1 are from estimating one home advantage parameter prior to COVID-19 and one afterwards. We then show the results of modelling home advantage separately for each season and show the results in Fig. 2 which reveal some interesting differences as discussed in “Results” section.

In (2), we see that the home team’s goal expectation is a linear combination of the home team’s offensive strength and the away team’s defensive strength as well as a constant home advantage. Conversely, the away team’s goal expectation is a linear combination of the away team’s offensive strength and the home team’s defensive strength with the home advantage parameter noticeably missing. There is no index for league because, although we use the same model consistently across each league, we fit a separate version for each league.

This model formulation results in the intercept representing the logarithm of the overall average of points scored with $exp(\beta _{sp}), exp(\omega _{sh[i]}),$ and $exp(\delta _{sa[i]})$ representing multiplicative increases or decreases to the average points scored to determine the expected points scored for an individual game. This can be seen by considering:

3 $$\begin{aligned} \begin{aligned} \text {log}(\mu _{i1})&= \gamma _{sp} + \beta _{sp} + \omega _{sh[i]} + \delta _{sa[i]} \\ \mu _{i1}&= \text {exp}(\gamma _{sp} + \beta _{sp} + \omega _{sh[i]} + \delta _{sa[i]}) \\ \mu _{i1}&= \text {exp}(\gamma _{sp})\times \text {exp}(\beta _{sp})\times \text {exp} (\omega _{sh[i]})\times \text {exp}(\delta _{sa[i]}) \end{aligned} \end{aligned}$$

For example, a home advantage parameter of $\beta = 0.25$ would result in multiplying the average points scored by $\text {exp}(0.25) \approx 1.28,$ which can be interpreted as an increase of about 28% in expected points scored by the home team in a game between teams with relative offensive and defensive strengths $\omega _{sh[i]}$ and $\delta _{sa[i]}$, respectively.

### Model fit in PyMC3

The models are fit using PyMC3, an open source probabilistic programming language that allows us to fit Bayesian models with their implementation of a gradient based Hamiltonian Monte Carlo (HMC) No U-Turn Sampler (NUTS)^25^. As in other previous work^10,18^, we use Bayesian modelling and fitting approaches to allow us to incorporate some prior baseline knowledge of parameters as well as better quantifying uncertainty in the interpretation of parameter estimates.

The Bayesian approach means we need to specify suitable prior distributions for all random parameters in the model. The prior distributions for parameters in our model are:

4 $$\begin{aligned} \begin{aligned} \gamma _{sp}&\sim \mathcal {N}(\theta ^*, \sigma ^{2*}) \\ \beta _{sp}&\sim \mathcal {N}(0, 1) \\ \lambda&\sim \text {Uniform}(0, 1000) \\ \omega _s&\sim \mathcal {N}(0, \sigma _{s\omega }) \\ \delta _s&\sim \mathcal {N}(0, \sigma _{s\delta }) \\ \sigma _{s\omega }&\sim \text {HalfNormal}(1) \\ \sigma _{s\delta }&\sim \text {HalfNormal}(1) \end{aligned} \end{aligned}$$

where $\theta ^*$ is the logarithm of the average points scored, and $\sigma ^{2*}$ is the logarithm of the variance of points scored, over the regular seasons and playoffs of the league being modelled. We note that we found $\gamma _{sp}$ fits close to $\theta ^*$ even when using a weakly informative prior, but we keep this formulation as it maintains the spirit of using prior information in Bayesian analysis. We allow $\lambda$ to potentially be large for instances where there is no overdispersion in the outcome variable because a large $\lambda$ results in a large $\alpha _{ij}$ which makes the Negative Binomial distribution become similar to a Poisson distribution.

The model is fit using PyMC3’s NUTS sampler using 4 chains of 2000 iterations with 1000 tune steps for a result of 8000 samples from 12,000 total draws. It is standard practice to check convergence with the ${\hat{R}}$ statistic from^31,32^. Each model fit produced ${\hat{R}}$ statistics of 1.00 with no divergences^26^. All code and data is available and can be found at: https://github.com/nicohiggs/home_advantage_covid19.

### Data

For each league we gathered data from the five most recent seasons spanning the years 2016–2020, both regular season and playoffs. For our model, for each game, we need to track the teams that are playing, which teams are home and away, their respective game point totals, which season the game occurred, and whether or not the game occurred in the playoffs or regular season.

The NHL data is sourced from Natural Stat Trick^33^. A typical NHL season consists of 82 games played by each team. Prior to the Vegas Golden Knights joining the league in 2017, there were 30 teams resulting in 1230 games per season. Since 2017 there are 1271 games played with 31 teams in the league. The playoffs consist of a bracket of 16 teams playing best-of-seven series, for an average of 80–90 games total. We note that the 2020 season was shortened to 1082 games due to stopping for the initial outbreak of the COVID-19 pandemic. The 2020 playoffs occurred inside the NHL bubble when play resumed, consisting of 6 games to determine positions 1–8 and 8 best-of-five series to determine positions 9–16 before beginning the usual playoff structure. This resulted in 129 games played in the NHL’s COVID-19 bubble.

The NBA data is sourced from the basketball-reference website^34^. The structure of the regular season and playoff schedules is similar to that of the NHL. A typical NBA season consists of 30 teams each playing 82 games for a total of 1230 games. The playoffs consist of a bracket of 16 teams playing best-of-seven series, for an average of 80–90 games total. Like the NHL, the 2020 NBA season was shortened to 971 games due to stopping for the initial outbreak of the COVID-19 pandemic. The 2020 playoffs occurred inside the NBA bubble when play resumed, consisting of 8 additional games for each of the top 22 teams to determine seeding of the top 16 teams before beginning the usual playoff structure. This resulted in 172 games played in the NBA’s COVID-19 bubble.

The MLB data is sourced from retrosheet^35^. A typical MLB season consists of 30 teams each playing 162 games for a total of 2430 games. The playoffs can be viewed as an 8 team bracket, but there are 4 “wildcard” teams that play two best-of-one games to determine the last two spots for the 8 teams that make the first round called the Division Series. The Division Series consists of best-of-five series to determine who moves on to the League Championship Series. The League Championship Series and the following World Series Championship consist of best-of-7 series to determine the winner. This playoff structure usually results in an average of 30–40 games. The 2020 COVID-19 restricted season reduced the number of scheduled games to 60 for each team. This change combined with cancellations due to outbreaks within teams reduced the total number of games to 898. The playoffs replaced the best-of-one wildcard round with best-of-three series involving all top 8 seeded teams. This resulted in a total of 52 playoff games. We note that the 2020 season saw some double-header games where teams switched home and away even though both games were played at the same stadium. We found this to have essentially no impact due these games making up a relatively small portion of total games (45/898) and to home advantage being so small in the MLB. We have reported the results with home and away defined as who batted last in each inning for all games.

The NFL data is sourced from the football-reference website^36^. A typical NFL season consists of 32 teams each playing 16 games for a total of 256 games. The playoffs usually consist of a bracket of the top 12 teams playing best-of-one games (the top 4 teams getting a first round bye) resulting in 11 games total. Although the 2020 season had restrictions on fan attendance, the regular season schedule did not change and the playoff set-up only slightly changed by expanding to consist of the top 14 teams (only the top 2 getting a first round bye) resulting in 13 games total. We exclude the Super Bowl as well as international site games from our analysis for consistency, as they are generally played at neutral sites.

## Supplementary Information

Supplementary Information 1.
