# Supplementary material for: Bayesian analysis of home advantage in North American professional sports before and during COVID-19
Source: Sci Rep. 2021 Jul 15;11:14521. doi: 10.1038/s41598-021-93533-w (PMC8282683; doi:10.1038/s41598-021-93533-w)
Supplement: Supplementary file 1 — Supplementary Information 1. [file 41598_2021_93533_MOESM1_ESM.pdf]

Bayesian Analysis of Home Advantage in North American Professional Sports Before and During COVID-19

Nico Higgs
Ian Stavness

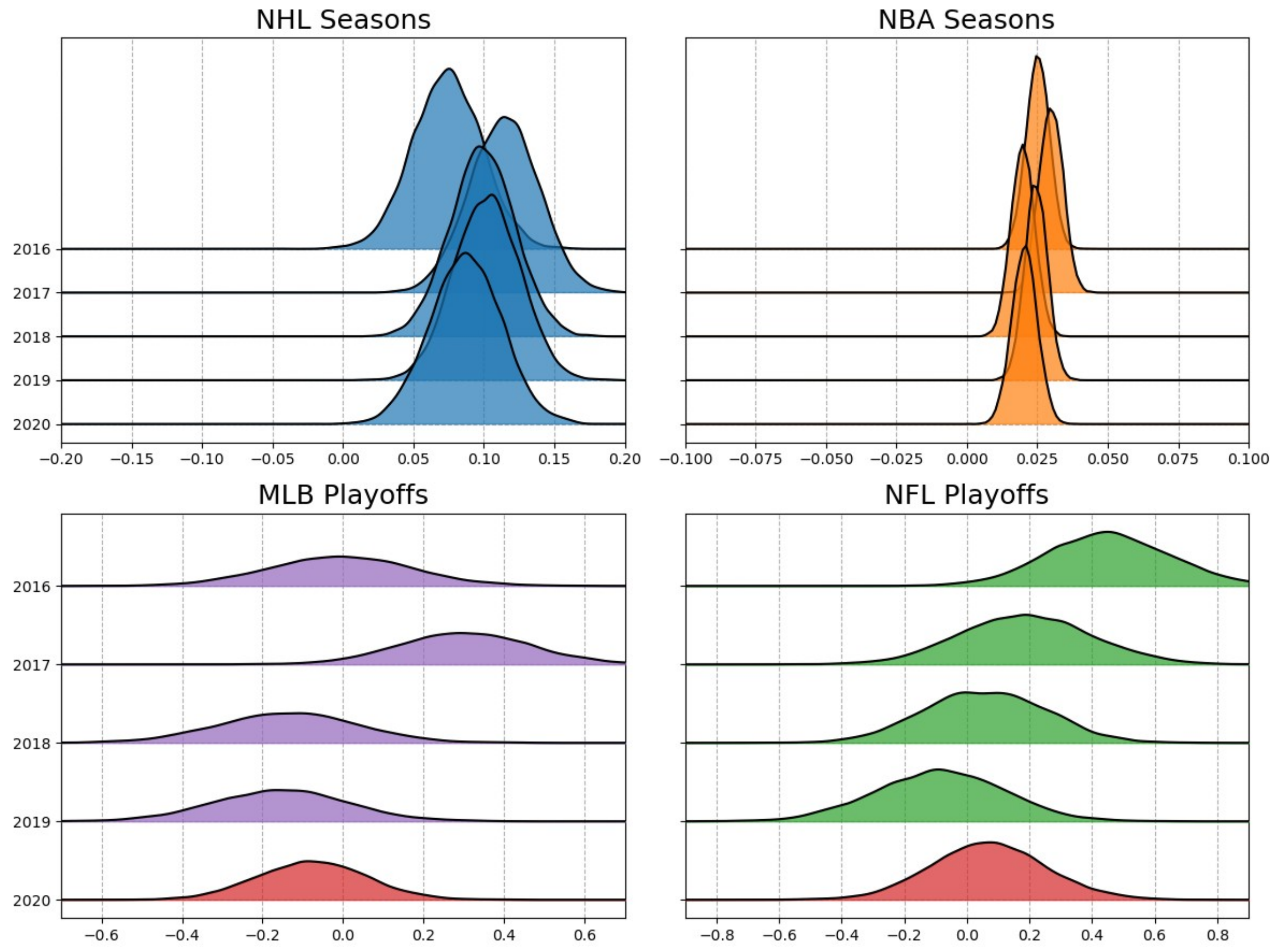

Supplementary Figure 1: Home advantage parameter estimates of NHL and NBA seasons, and MLB and NFL playoffs. COVID-19 restricted seasons are shown in red. The NHL and NBA 2020 seasons were completed prior to the COVID-19 lockdown and thus had no restrictions.

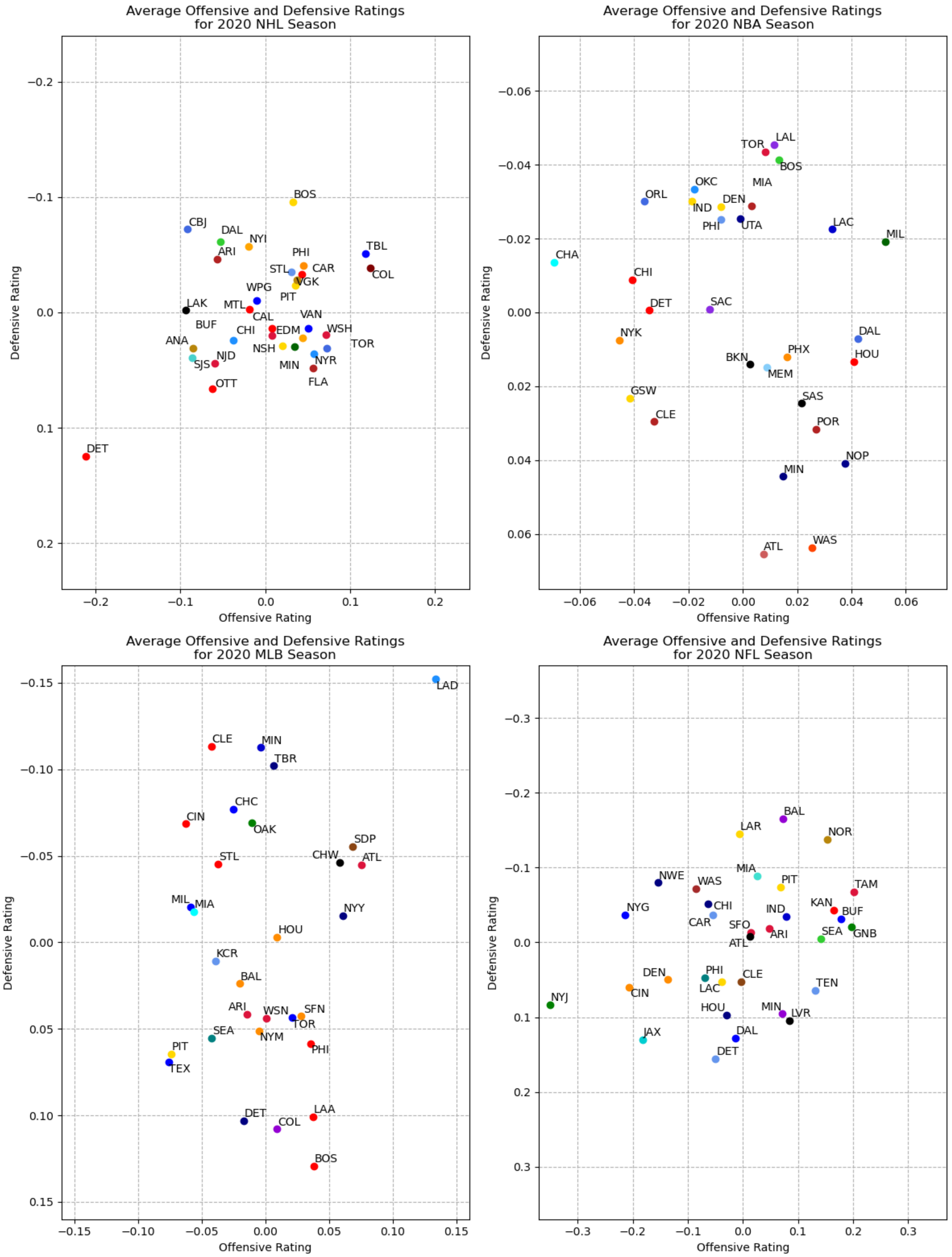

Supplementary Figure 2: Average team ratings for each league for the 2020 season.
